# Maternal Folic Acid Supplementation during Pregnancy Prevents Hepatic Steatosis in Male Offspring of Rat Dams Fed High-Fat Diet, Which Is Associated with the Regulation of Gut Microbiota

**DOI:** 10.3390/nu15224726

**Published:** 2023-11-08

**Authors:** Huaqi Zhang, Yutong Wang, Xinyu Zhang, Li Zhang, Xuenuo Zhao, Yan Xu, Peng Wang, Xi Liang, Meilan Xue, Hui Liang

**Affiliations:** 1 Department of Nutrition and Food Hygiene, School of Public Health, Qingdao University, Qingdao 266071, China; huaqi_erin@163.com (H.Z.); wyt687199@126.com (Y.W.); zzzzzxy1022@126.com (X.Z.); zhangli200513@163.com (L.Z.); nuo0109996@163.com (X.Z.); xy2514580989@163.com (Y.X.); wpeng@qdu.edu.cn (P.W.); liangxi6029@163.com (X.L.); 2 Department of Biochemistry and Molecular Biology, Basic Medical College, Qingdao University, Qingdao 266071, China; snowml@126.com

**Keywords:** folic acid, offspring, hepatic steatosis, intestinal barrier, endotoxin leakage, gut microbiota

## Abstract

Maternal dietary patterns during pregnancy have been demonstrated to impact the structure of the gut microbiota in offspring, altering their susceptibility to diseases. This study is designed to elucidate whether the impact of folic acid supplementation during pregnancy on hepatic steatosis in male offspring of rat dams exposed to a high-fat diet (HFD) is related to gut–liver axis homeostasis. In this study, female rats were administered a HFD and simultaneously supplemented with 5 mg/kg folic acid throughout their pregnancy. Histopathological examination showed that folic acid supplementation effectively ameliorated hepatic lipid accumulation and inflammatory infiltrate in male offspring subjected to a maternal HFD. Maternal folic acid supplementation reduced the abundance of *Desulfobacterota* and the *Firmicutes*/*Bacteroidota* (F/B) ratio in male offspring. The expression of tight junction proteins in the colon was significantly upregulated, and the serum LPS level was significantly reduced. Furthermore, there was a notable reduction in the hepatic expression of the TLR4/NF-κB signaling pathway and subsequent inflammatory mediators. Spearman’s correlation analysis revealed significant associations between hepatic inflammation-related indices and several gut microbiota, particularly *Desulfobacterota* and *Lactobacillus*. With a reduction in hepatic inflammation, the expression of PPAR-α was upregulated, and the expression of SREBP-1c and its downstream lipid metabolism-related genes was downregulated. In summary, folic acid supplementation during pregnancy modulates gut microbiota and enhances intestinal barrier integrity in male offspring of HFD dams. This helps reduce the LPS leakage and suppress the expression of TLR4/NF-κB pathway in the liver, thereby improving lipid metabolism disorders, and alleviating hepatic steatosis.

## 1. Introduction

The developmental origins of health and disease (DOHaD) hypothesis proposes that increased susceptibility to chronic disease is partly shaped by early-life nutritional disorders, which has been supported by evidence from both epidemiological investigations and animal studies [1,2,3]. A maternal high-fat diet (HFD) during pregnancy has been observed to have detrimental effects on various aspects of health in offspring. Studies have revealed that a maternal HFD during pregnancy can impact bone development, mesolimbic function, and social behaviors in offspring [4,5,6]. Furthermore, a large number of studies have highlighted the unfavorable outcomes of maternal HFD on lipid metabolism in offspring. These effects include significantly elevated serum triglyceride (TG) levels and the occurrence of hepatic lipid accumulation in weanling offspring, with persistent effects in adulthood [7,8,9]. Although supplementation with active substances such as lycopene and betaine during pregnancy can mitigate the effects of maternal HFD on lipid metabolism disorder in offspring in animal studies [10,11], these active substances are treated with caution during human pregnancy due to safety concerns.

Folic acid is a water-soluble vitamin, and its supplementation is one of the focuses of periconceptional health care for women in childbearing age owing to its role in preventing fetal neural tube abnormalities [12]. Over the past few years, several population and animal studies have demonstrated that folic acid supplementation during pregnancy remedied lipid metabolism disorder in offspring. This effect is achieved by inducing alterations in the DNA methylation of PPAR-α and PPAR-γ [13,14]. Administering methyl donors, which encompass folic acid, to dams subjected to high-fat sucrose diet during lactation confers protection upon their offspring against the accumulation of fat in liver via DNA methylation, fatty acid oxidation, and insulin resistance [15]. However, DNA methylation is not sufficient to explain the programming effects mediated by folic acid, and other potential mechanisms still need to be explored.

Evidence indicates that folic acid possesses the capability to improve obesity, alcoholic liver injury and hyperuricemia by stabilizing the gut microbiota [16,17,18], which provides new clues to our study. Numerous studies have demonstrated that the maternal diet significantly influences the establishment and development of the offspring’s gut microbiota, subsequently impacting the disease susceptibility. For example, maternal aspartame and stevia exposure altered the microbiota in offspring, which promoted obesity and impaired glucose tolerance [19]. Metformin treatment in HFD dams restored the gut microbiota composition in offspring and improved male offspring’s metabolic phenotype [20]. However, the efficacy of folic acid in mitigating the maternal HFD-induced disorders of the gut microbiota in offspring, thereby alleviating hepatic steatosis via the gut–liver axis, is unclear.

In this study, we gave maternal rats HFD with 5 mg/kg folic acid, focusing on male offspring hepatic steatosis at weaning and exploring the mechanism through the gut–liver axis.

## 2. Materials and Methods

### 2.1. Animals

Eight-week-old male and female Sprague Dawley rats weighing 250 ± 10 g were purchased from Vital River (Beijing, China). The animals were kept in the animal center of Qingdao University with standardized environment (21 ± 2 °C, 50% ± 10% relative humidity, and 12 h light/dark cycle). The rats were provided with freely available food and water. All experimental protocols were approved by the Ethics Committee Medical College of Qingdao University, and we adhered to the institution’s guidelines of laboratory animals (approval number: QDU-AEC-2023341).

### 2.2. Experimental Design

**Figure 1:**
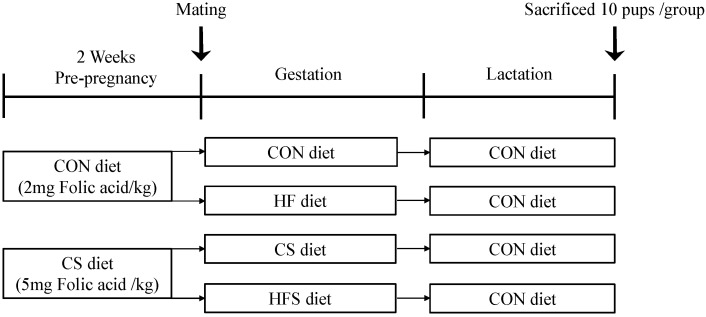
Schedule of the experimental and dietary intervention protocol.

After one week of adaptation, females were randomly divided into two groups. The control group (CON) was given 2 mg folic acid/kg diet. The control with folic acid supplementation group (CS) was given 5 mg folic acid/kg diet. Male rats designated for breeding were provided with an identical diet to that of the CON females before mating. After 2 weeks, the females were mated with males (three females and one male per cage). Pregnancy was confirmed by the detection of sperm in the vaginal smear. On the day when pregnancy was detected, the dams were then further divided randomly into two groups within the original divisions to a control (3850 kcal/kg; 10% kcal from fat, 20% kcal from protein, and 70% kcal from carbohydrate) or high fat (5243 kcal/kg; 60% kcal from fat, 20% kcal from protein, and 20% kcal from carbohydrate) diet. The high-fat group (HF) and a high-fat with folic acid supplementation group (HFS) were established. At birth, within each group, every litter was reduced to 6 male pups to mitigate variations in milk provision. All dams were fed with a CON diet during lactation. The specific dietary compositions are outlined in Table S1. The schedule of the experimental and dietary intervention protocol is portrayed in Figure 1.

The body weight of offsprings were measured weekly. At 3 weeks of age, we randomly selected one pup from each litter, and a total of 10 pups were selected for each group. After fasting for 10 h, the pups were anesthetized, followed by blood, liver, colon and colon content sample collection. The collected blood was centrifuged (3000 rpm, 10 min, and 4 °C) after being left for 30 min to clot, and the obtained serum was promptly stored at −80 °C. The liver was promptly excised and weighed for the calculation of the liver index (liver index % = liver weight/body weight × 100%) accurately. A portion of the liver and colon tissues were fixed in formaldehyde (10%) solution for histopathological analysis. Remaining liver and colon tissues were rapidly frozen using liquid nitrogen and subsequently stored at −80 °C.

### 2.3. Measurement of Metabolites in Serum

The fasting serum levels of glucose (GLU), insulin (INS), TG, total cholesterol (TC), high-density lipoprotein cholesterols (HDL-C), and low-density lipoprotein cholesterols (LDL-C) were measured using an AU5400 automatic biochemical analyzer (Beckman, Los Angeles, CA, USA). The homeostatic model assessment of insulin resistance (HOMA-IR) is calculated as follows: fasting serum glucose (mmol/L) × serum insulin (mIU/L)/22.5. Serum lipopolysaccharide (LPS) concentration was measured using the Endpoint Chromogenic Endotoxin Detection LAL Kit.

### 2.4. Hepatic Biochemical Analysis

Hepatic TG, TC, interleukin-1β (IL-1β), interleukin 6 (IL-6) and tumor necrosis factor-α (TNF-α) levels were assessed by ELISA using commercial kits (Jiancheng Bioengineering Institute, Nanjing, China) according to the manufacturer’s instructions.

### 2.5. Oral Glucose Tolerance Test (OGTT)

At 3 weeks of age, male offsprings were given sterile glucose by gavage (50% glucose solution, 2 g/kg body weight) after fasting for 10 h. Blood samples were collected through the tail vein at 0, 15, 30, 60, and 120 min. Glucose concentrations were quantified using the ACCU-CHEK Mobile (Roche, Basel, Switzerland).

### 2.6. Histopathological Examination

The liver and colon samples were fixed using a 10% paraformaldehyde solution and subsequently embedded in paraffin prior to being sectioned. Hematoxylin and eosin (H&E) staining was then conducted on the liver sections to evaluate hepatic steatosis. Histopathological alterations in the liver and colon sections were observed BX60 light microscope (Olympus, Tokyo, Japan). The analysis of the number of lipid droplets followed previous studies [21,22].

### 2.7. Western Blotting

Western blotting was conducted in accordance with previously established protocols [23]. The protein content was quantified using the BCA Protein Assay Kit (Beyotime, Zhenjiang, China). Proteins were separated by 10% polyacrylamide gels and subsequently transferred onto PVDF membranes (Millipore, MA, USA) and then subjected to an overnight incubation at 4 °C with primary antibodies against Notch1, NF-κB, ACC, FAS, Occludin (Cell Signaling Technology, MA, USA), TLR4, IκBα, p-IκBα, PPAR-α, SREBP-1c, ZO-1, Claudin1, β-actin and Lamin B (Santa Cruz, CA, USA). The membranes were washed three times with TBST, then exposed to the corresponding secondary antibody for 2 h at 37 °C. β-Actin and lamin B served as internal control for cytosolic and nuclear proteins.

### 2.8. DNA Extraction and 16S rRNA Gene Sequencing for Microbiome Analysis

Seven samples from each group were randomly selected for 16S rRNA gene sequencing. Sequencing methods followed previous studies [17,24]. Specifically, DNA was extracted using the DNA Kit from Tiangen Biotech (Beijing, China). Forward primer 338F: 5′-ACTCCTACGGAGGCAGCA-3′ and reverse primer 806R: 50-GGACTACHVGGGTWTCTAAT-3′ was used to amplify the variable region 3–4 (V3–V4). The PCR products were subjected to agarose gel electrophoresis, and DNA quantification was performed using a NanoDrop 2000 spectrophotometer (Thermo Fisher Scientific, Waltham, MA, USA). For the constructed library, sequencing was performed using Illumina NovaSeq6000 platform (San Diego, CA, USA). The raw data were spliced using USEARCH (version 10). To obtain high-quality tag sequences, chimeras were eliminated using UCHIME (version 8.1). Sequences were clustered using USEARCH to filter out OTUs at a 97% similarity threshold. The alpha diversity and beta diversity were analyzed by using QIIME. In addition, the LDA effect size (LEfSe) algorithm was used to analyze the differential abundance of each group of gut microbiota. The logarithmic LDA score was set at 4.0 to determine significant differences.

### 2.9. Statistical Analysis

Data analysis and graphical design were performed using SPSS 26.0 (SPSS, Chicago, IL, USA) and GraphPad Prism 9 (GraphPad Software, Boston, MA, USA). All data were presented as mean ± SD. Group differences were estimated using one-way analysis of variance (ANOVA). Tukey’s post hoc test was used to analyze the difference between two groups. The significance in gut microbiota analysis was assessed using the Kruskal–Wallis ranking test. Correlations between gut microbiota and biochemical indices and inflammatory factors were tested using Spearman’s correlation analysis. The correlation heat maps were produced using the R software package (version 3.6.3). A significance level of *p* < 0.05 was regarded as statistically significant.

## 3. Results

### 3.1. Maternal Food Consumption during Pregnancy and Male Pups’ Body Weight, Liver Weight

During pregnancy, there was no significant difference in the maternal average daily food consumption among the four groups. The maternal average daily energy intake in the HF group (83.75 ± 8.27 kcal/d) and the HFS group (84.15 ± 8.32 kcal/d) were both significantly higher compared to the CON group (62.92 ± 6.89 kcal/d). The maternal average daily folic acid intake was significantly higher in the CS group (82.88 ± 9.24 µg/d) and the HFS group (80.25 ± 7.93 µg/d) compared to the CON group (32.69 ± 3.58 µg/d) and the HF group (31.95 ± 3.15 µg/d).

**Figure 2:**
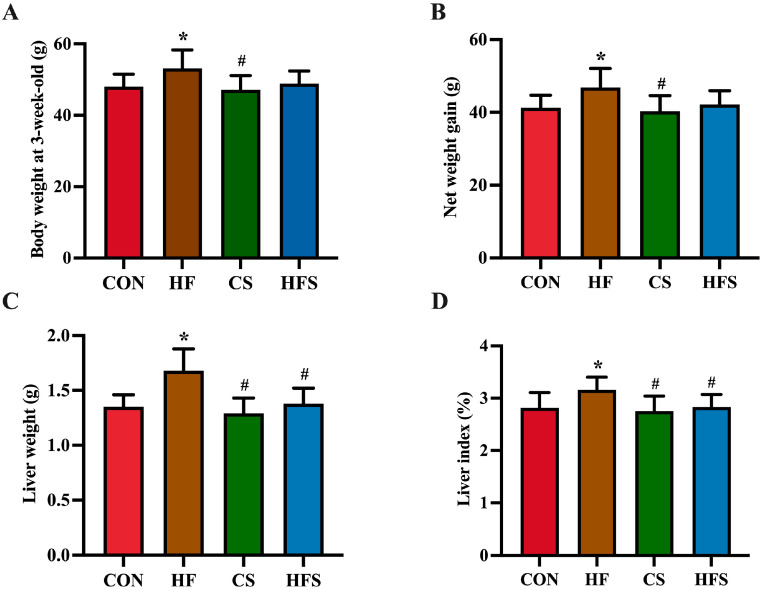
Body weight and liver weight. (**A**) Body weight at 3-week-old; (**B**) net weight gain; (**C**) liver weight; and (**D**) liver index. Data are presented as mean ± SD (*n* = 10). * *p* < 0.05 vs. CON; ^#^ *p* < 0.05 vs. HF.

In male offspring, no significant difference was found in body weight from birth to the first two weeks. At 3 weeks old, a significant increase in body weight was found in the HF group compared to the CON group (*p* < 0.05; Figure 2A), whereas the body weight in the HFS group was not significantly different from that of the CON group. The net weight gain at 3 weeks old exhibited a significant increase in the HF group compared to the CON group (*p* < 0.05; Figure 2B). There was no significant difference in net weight gain between the HFS group and the CON group. The HF group displayed a significantly higher liver weight and liver index compared to the CON group, whereas these parameters in the HFS group were significantly normalized (*p* < 0.05; Figure 2C,D).

### 3.2. Effects of Maternal Folic Acid Supplementation on Serum Parameters and Glucose Tolerance in Male Offspring

As demonstrated in Table 1, the serum concentrations of TG, LDL-C and LPS were significantly elevated in the HF group compared to the CON group (*p* < 0.05). The HFS group exhibited significantly reduced levels of TG and LPS compared to the HF group.

Maternal HFD during pregnancy resulted in a significant increase in GLU and HOMA-IR in male offspring. In the HFS group, GLU showed a significant decrease compared to the HF group (*p* < 0.05). There was no significant difference in HOMA-IR between the HFS group and the CON group. No significant difference was found in serum INS levels among the four groups.

**Figure 3:**
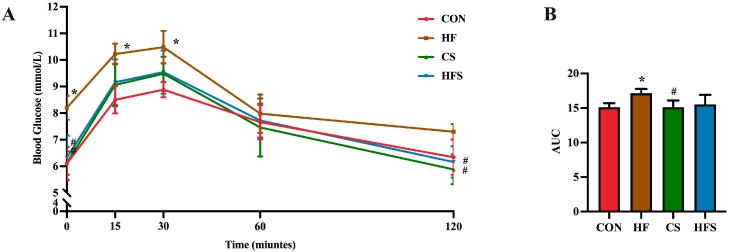
Effects of folic acid on glucose tolerance in male offspring. (**A**) Blood glucose analysis of glucose tolerance test; (**B**) AUC of blood glucose. Data are presented as mean ± SD (*n* = 5). * *p* < 0.05 vs. CON, ^#^ *p* < 0.05 vs. HF.

During OGTT, as shown in Figure 3A, the level of blood glucose in the HF group exhibited a significant increase at 0, 15, and 30 min, whereas the CS and HFS group displayed significantly lower blood glucose levels at 0 and 120 min compared to the HF group (*p* < 0.05). In addition, the area under the curve (AUC) in the HF group showed a significant increase compared to that in the CON group (*p* < 0.05; Figure 3B), whereas the AUC in the HFS group was not significantly different from that in the CON group.

### 3.3. Effects of Maternal Folic Acid Supplementation on Hepatic Histopathology and Parameters in Male Offspring

**Figure 4:**
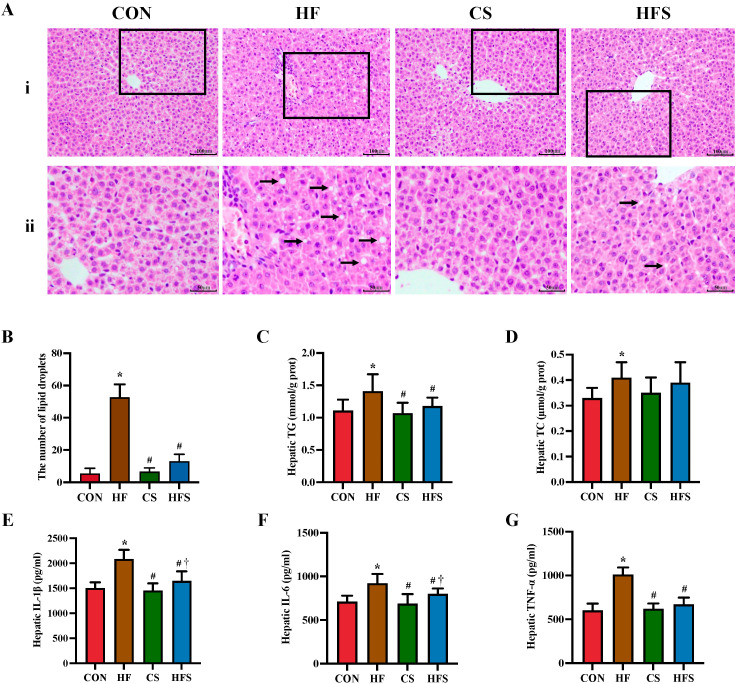
Hepatic pathological changes, lipid profiles, and inflammatory cytokines. (**A**) (i) row shows H&E staining of hepatic histopathology (×200); representative pathological visual fields were shown in the inset boxes; (ii) row shows an enlarged version of the inset boxes in i row; lipid droplets were indicated by black arrows; (**B**) the number of lipid droplets in fields (*n* = 5); (**C**) hepatic TG; (**D**) hepatic TC; (**E**) hepatic IL-1β; (**F**) hepatic IL-6; and (**G**) hepatic TNF-α. Data are presented as mean ± SD (*n* = 10). * *p* < 0.05 vs. CON, ^#^ *p* < 0.05 vs. HF, ^†^ *p* < 0.05 vs. CS.

As shown in Figure 4A, H&E staining revealed that the HF group had disorganized hepatic cords with inflammatory cell infiltration. The number of lipid droplets was significantly higher in the HF group than in the CON group (Figure 4B). Compared with the HF group, the hepatic cords in the HFS group were arranged radially, without obvious inflammatory infiltrate. The number of hepatic lipid droplets was significantly lower in the HFS group than in the HF group. The HF group displayed significantly higher hepatic TG levels compared to the CON group, while the HFS group had significantly more normalized hepatic TG levels (*p* < 0.05; Figure 4C). In comparison to the CON group, the HF group demonstrated a significant increase in hepatic levels of TNF-α, IL-1β, and IL-6, whereas hepatic TNF-α, IL-1β, and IL-6 levels in the HFS group were significantly lower than those in the HF group (*p* < 0.05; Figure 4E–G).

### 3.4. Effects of Maternal Folic Acid Supplementation on Colon Histopathology and the Tight Junction Protein Expression Levels in Male Offspring

**Figure 5:**
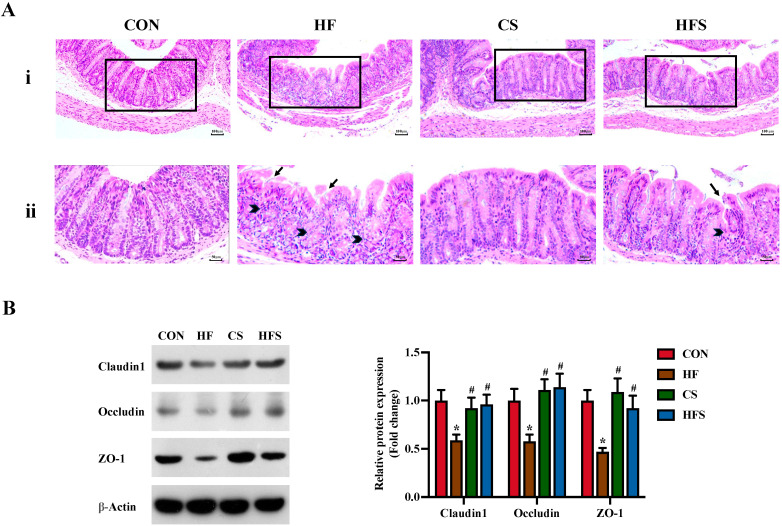
Colon histopathology and the tight junction protein expression levels in male offspring. (**A**) the (i) row shows colon histopathology assessed using H&E staining (100×); representative pathological visual fields were shown in the inset boxes; the (ii) row shows an enlarged version of the inset boxes in the (i) row; damaged mucosal surface or disordered glandular arrangement were denoted as black arrows and black arrowheads, respectively; (**B**) the expression of Claudin 1, Occludin, and ZO-1 protein in the colon detected using Western blot. Data are presented as mean ± SD (*n* = 3). * *p* < 0.05 vs. CON, ^#^ *p* < 0.05 vs. HF.

In the H&E staining, the colon mucosa mechanical barrier in the HF group was compromised, characterized by damaged mucosal surface integrity and disordered glandular arrangement. In contrast, in the HFS group, the mucosal integrity was restored, and the glandular arrangement was relatively normal in shape (Figure 5A).

We detected tight junction proteins in the colon and observed that the expression levels of tight junction proteins (Claudin 1, Occludin, and ZO-1) were significantly reduced in the HF group, while the expression of these proteins significantly recovered in the HFS group (*p* < 0.05; Figure 5B).

### 3.5. Maternal Folic Acid Supplementation Regulates Hepatic TLR4/NF-κB Signaling Pathway and Lipid Metabolism in Male Offspring

**Figure 6:**
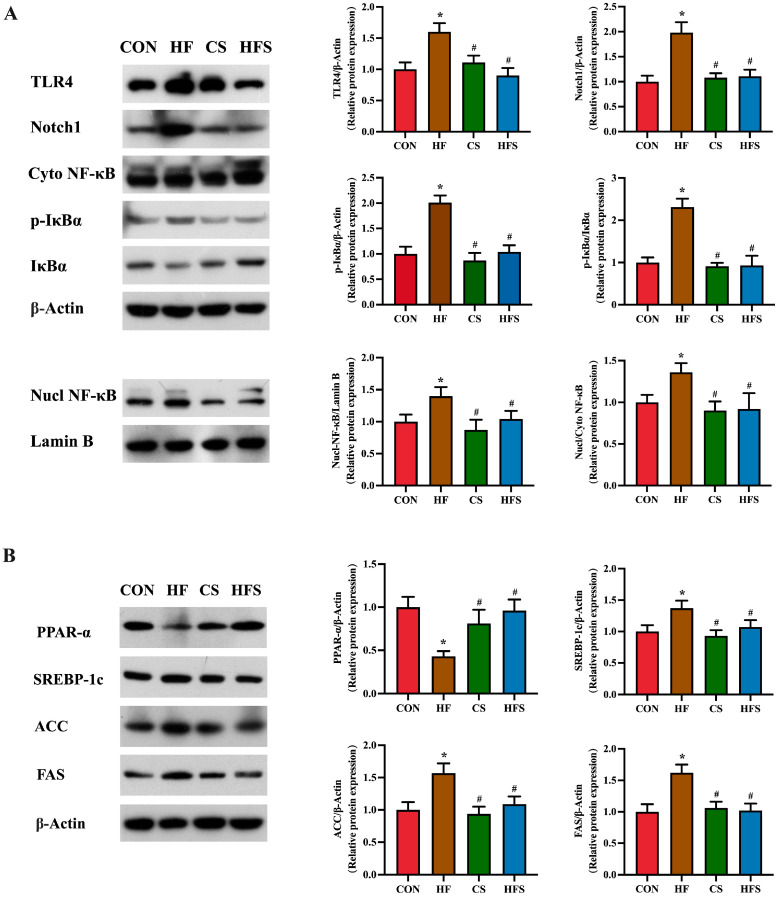
TLR4/NF-κB signaling pathway and lipid metabolism in liver. (**A**) The expression of TLR4, Notch1, p-IκBα, p-IκBα/IκBα, Nucl NF-κB, and Cyto NF-κB protein levels in liver, detected using Western blot; (**B**) the expression of PPAR-α, SREBP-1c, ACC, and FAS protein levels in liver, detected using Western blot. Data are presented as mean ± SD (*n* = 3). * *p* < 0.05 vs. CON, ^#^ *p* < 0.05 vs. HF.

In comparison to the CON group, the HF group exhibited notably elevated expression levels of TLR4, Notch1, p-IκBα, as well as NF-κB (*p* < 0.05). A significant reduction in the expression levels of these proteins was observed in the HFS group when compared to the HF group (*p* < 0.05, Figure 6A).

As shown in Figure 6B, compared with the CON group, PPAR-α expression levels were significantly downregulated, and SREBP-1c, ACC, and FAS expression levels were significantly upregulated in the HF group. Compared with the HF group, the HFS group had significantly increased PPAR-α expression and significantly decreased SREBP-1c, ACC and FAS expressions.

### 3.6. Effects of Maternal Folic Acid Supplementation on Gut Microbiota in Male Offspring

#### 3.6.1. Rarefaction Curve and the Numbers of OTUs

**Figure 7:**
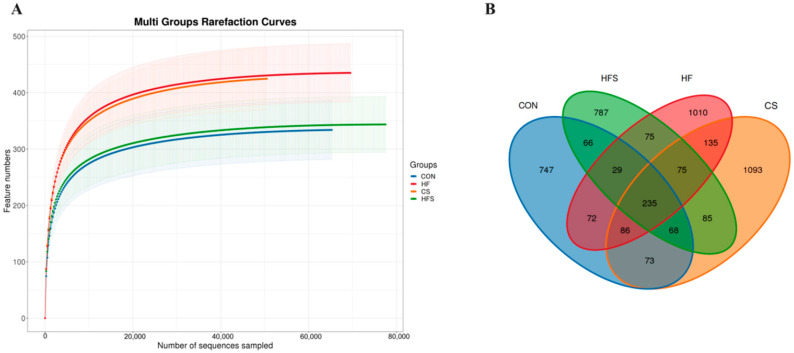
The rarefaction curves and the Venn diagram for each sample in male offspring. (**A**) Bacterial rarefaction curves; (**B**) Venn diagram.

Good’s coverage index of each group based on OTU abundance was >99.99%. The sparsity curve flattens out as the number of sequences in the sample increases, indicating that the sequencing quantity and depth meet the requirements for further analysis (Figure 7A). The Venn diagram showed that 235 gut microbes were common in the four groups, and there were 747 exclusive gut microbes in the CON group, 1010 exclusive gut microbes in the HF group, 1093 exclusive gut microbes in the CS group, and 787 exclusive gut microbes in the HFS group (Figure 7B).

#### 3.6.2. Alpha Diversity Analysis

**Figure 8:**
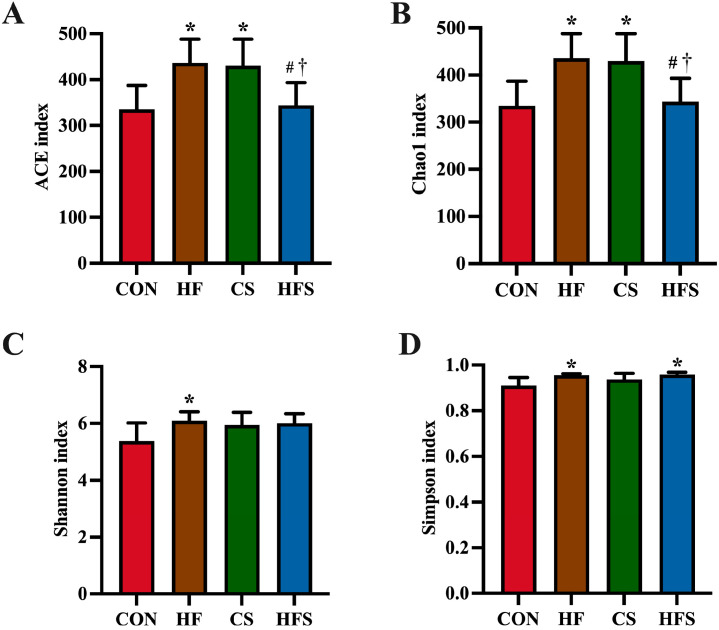
Alpha diversity analysis of male offspring gut microbiota. ACE (**A**), Chao 1 (**B**), Shannon (**C**) and Simpson index (**D**). Data are presented as mean ± SD (*n* = 7). * *p* < 0.05 vs. CON; ^#^ *p* < 0.05 vs. HF; ^†^ *p* < 0.05 vs. CS.

The findings revealed significant differences in the ACE and Chao 1 index with markedly elevated in the HF and CS groups in comparison to the CON group. Conversely, significantly normalized the ACE index and the Chao 1 index were exhibited in the HFS group (*p* < 0.05; Figure 8A,B). The Shannon index was significantly lower in the HF compared with the CON group, whereas the Shannon index in the HFS group was not significant different from the CON group (*p* < 0.05; Figure 8C). In comparison to the CON group, the Simpson index was remarkably elevated in the HF and HFS groups (*p* < 0.05; Figure 8D).

#### 3.6.3. Beta Diversity Analysis

**Figure 9:**
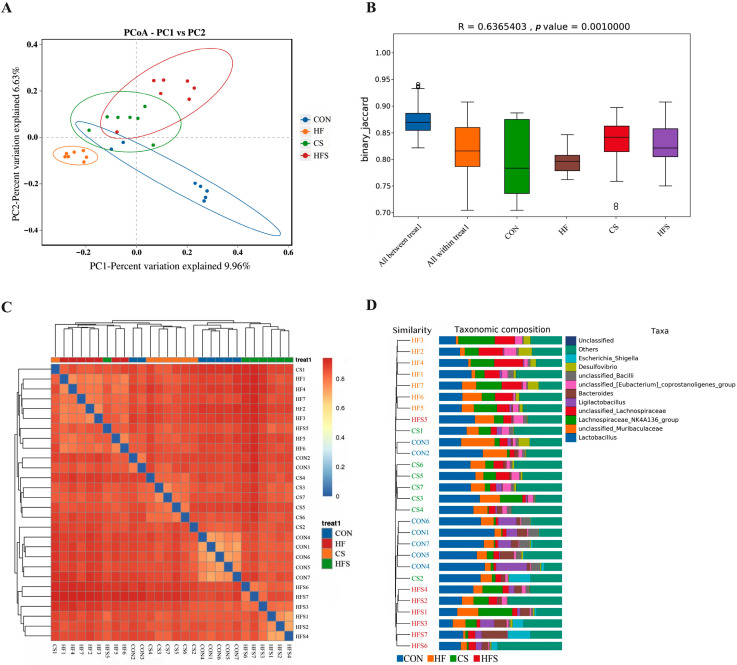
Beta diversity analysis of male offspring gut microbiota. (**A**) Principal coordinate analysis (PCoA) of beta diversity among four groups; (**B**) analysis of similarities (ANOSIM) assessing gut microbiota differences among male offspring; (**C**) beta diversity heatmap representing offspring gut microbiota variation; and (**D**) unweighted pair-group method with arithmetic mean (UPGMA) clustering of male offspring microbiota.

The gut microbiota communities among the four groups exhibited distinct differences, as evidenced by the principal coordinate analysis (PCoA). As shown in Figure 9A, the gut microbiota community of the HF group exhibited a distinct separation from the CON group, while the gut microbiota community of the HFS group displayed more similarity to the CON group. Furthermore, the differences among the groups were more pronounced than that within the groups (r = 0.637 and *p* = 0.001; Figure 9B). The community compositions of the CS and HFS groups were more similar to the CON group when compared with the community composition between the HF group and the CON group (Figure 9C,D).

#### 3.6.4. Microbial Phylum Level Composition

**Figure 10:**
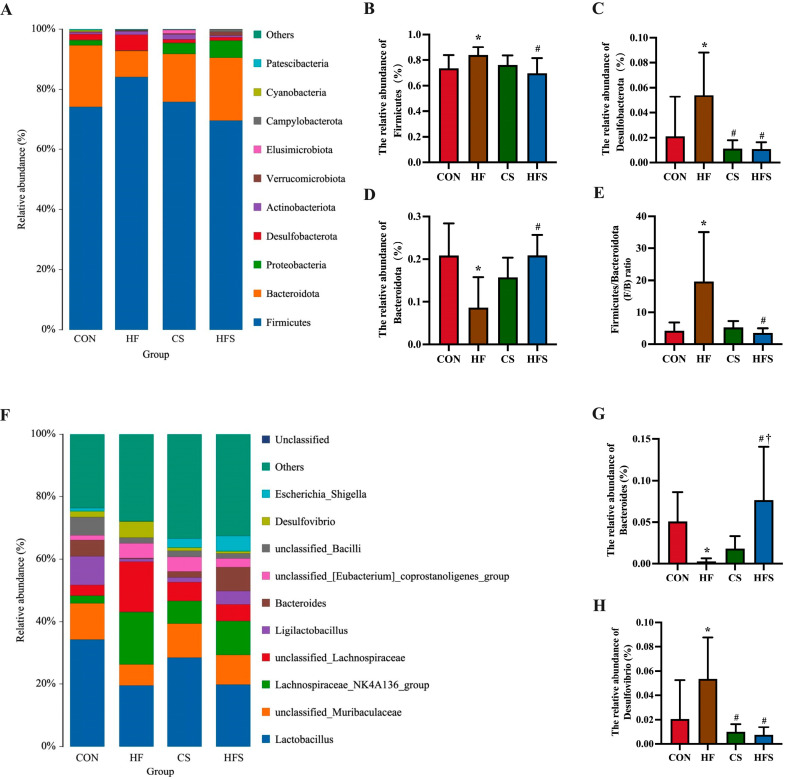
The gut microbiota structure in male offspring. (**A**) Microbial phylum composition profile among four groups; (**B**) relative abundance of *Firmicutes*; (**C**) relative abundance of *Desulfobacterota*; (**D**) relative abundance of *Bacteroidota*; (**E**) the *Firmicutes*/*Bacteroidota* (F/B) ratio; (**F**) microbial genus composition profile among four groups; (**G**) abundance of *Bacteroides*; and (**H**) abundance of *Desulfovibrio*. Data are presented as mean ± SD (*n* = 7). * *p* < 0.05 vs. CON, ^#^ *p* < 0.05 vs. HF, ^†^ *p* < 0.05 vs. CS.

At the phylum level, the predominant constituents of the gut microbiota included *Firmicutes*, *Bacteroidota*, *Proteobacteria*, *Desulfobacterota*, and *Actinobacteriota*. As shown in Figure 10B,C, the abundance of *Firmicutes* and *Desulfobacterota* had a significant increase in the HF group compared with the CON group, while in the HFS group, it displayed a marked reduction compared with the HF group (*p* < 0.05). The *Bacteroidota* abundance was significantly lower in the HF group when compared with the CON group; conversely, it was significantly higher in the HFS group compared with the HF group (*p* < 0.05; Figure 10D). The *Firmicutes*/*Bacteroidota* (F/B) ratio exhibited a significant increase in the HF group when compared with the CON group, whereas it exhibited a significant reduction in the HFS group in comparison to the HF group (*p* < 0.05; Figure 10E).

#### 3.6.5. Microbial Genus-Level Composition

At the genus level, the gut microbiota in offspring consisted mainly of *Lactobacillus*, *unclassified_Muribaculaceae*, *Lachnospiraceae_NK4A136_group*, *unclassified_Lachnospiraceae*, and *Ligilactobacillus*. The abundance of *Bacteroides* was significantly lower in the HF group than in the CON group. The HFS group had a significantly higher abundance of *Bacteroides* compared with the HF group (*p* < 0.05; Figure 10G). In comparison to the CON group, the HF group showed a significantly higher abundance of *Desulfovibrio*, whereas the HFS group displayed a significantly lower abundance of *Desulfovibrio* compared with the HF group (*p* < 0.05; Figure 10H).

#### 3.6.6. LEfSe Analysis

**Figure 11:**
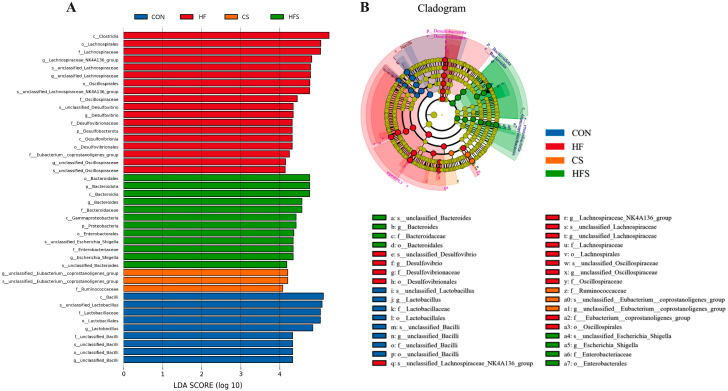
LEfSe analysis. (**A**) Histogram of the LDA scores; (**B**) LEfSe analysis evolutionary branching diagram of gut microbiota.

The results of LEfSe analysis showed that 42 OTUs differed among the four groups. The prevailing bacterium in the CON group was mainly *c_Bacilli*, the HF group was mainly *c_Clostridia* and *o_Lachnospirales*, the CS group was mainly *g_unclassified Eubacterium_coprostanoligenes_group* and the HFS group was mainly *Bacteroidales* (Figure 11).

#### 3.6.7. Spearman’s Correlations between Hepatic Inflammation-Related Indices and Gut Microbiota

**Figure 12:**
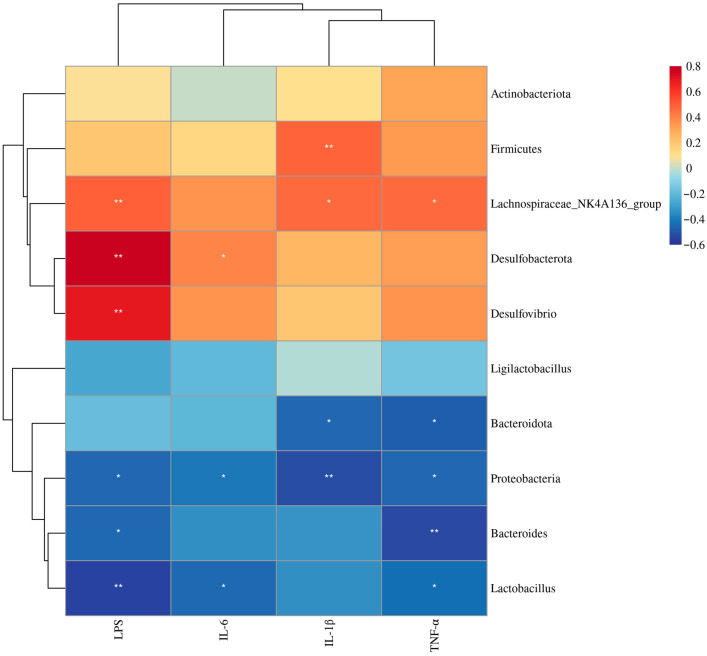
Spearman’s correlations between hepatic inflammation-related indices and gut microbiota in male offspring, including LPS, IL-6, IL-1β, and TNF-α. Data are presented as mean ± SD (*n* = 7). * *p* < 0.05, ** *p* < 0.01.

As illustrated in Figure 12, LPS showed a significant positive correlation with the *Desulfobacterota*, *Desulfovibrio*, and *Lachnospiraceae_NK4A136_group*, while exhibiting a significant negative correlation with *Bacteroides*, *Lactobacillus*, and *Proteobacteria*. IL-6 levels correlated positively with *Desulfobacterota*, but correlated negatively with *Lactobacillus* and *Proteobacteria*. IL-1β levels correlated positively with *Firmicutes* and *Lachnospiraceae_NK4A136_group*, but correlated negatively with *Proteobacteria* and *Bacteroidota*. TNF-α levels displayed a significant positive correlation with *Lachnospiraceae_NK4A136_group*, while it was correlated negatively with *Bacteroides*, *Bacteroidota*, *Lactobacillus*, and *Proteobacteria*.

## 4. Discussion

Maternal folic acid supplementation during pregnancy demonstrated a protective effect against hepatic steatosis in male offspring of HFD-exposed dams. The alterations detected in the composition and abundance of gut microbiota in male offspring may be relevant to maternal folic acid supplementation during pregnancy, subsequently enhancing gut barrier integrity. The enhancement of intestinal barrier function prevents the influx of endotoxins into the liver with the blood, thereby inhibiting the TLR4/NF-κB pathway and mitigating lipid metabolism disorders.

HFD is a prevalent unfavorable dietary pattern during pregnancy [25]. Maternal HFD has been demonstrated to have adverse effects on lipid metabolism in offspring and promote the onset of hepatic steatosis [20,26,27]. Hepatic steatosis serves as the beginning of non-alcoholic fatty liver disease (NAFLD), often accompanied by dysregulated blood and hepatic lipid levels [28]. Histopathological examination is widely recognized as the gold standard for its diagnosis [29]. In the present study, we administered HFD to dams during the entire pregnancy and observed significantly high serum and hepatic TG levels in their 3-week-old male offspring. Histopathological examination further revealed the presence of hepatic lipid accumulation and inflammatory infiltration in the offspring of HFD dams. The findings above indicate that maternal HFD during pregnancy promotes the development of hepatic steatosis in 3-week-old offspring. Notably, hepatic steatosis during childhood has the potential to advance into end-stage liver disease during adulthood, severely affecting quality of life [30]. Whether active dietary intervention during pregnancy is effective in mitigating the promoting effect of HFD on hepatic steatosis in offspring deserves further investigation.

Folic acid, known as a water-soluble vitamin, is effective in preventing neural tube abnormalities [31]. In recent years, it has been discovered that folic acid supplementation during pregnancy, either as a standalone intervention or in combination with other substances, can mitigate the adverse impacts of an unfavorable intrauterine environment on offspring, including lipid metabolism, nephrogenesis, and neurogenesis [32,33,34]. In these studies, 5 mg/kg folic acid supplementation, based on the upper tolerable intake limit for pregnant women (1000 μg/day), has been widely used in rodents [35]. In this study, the average litter size of dams among the four groups showed no significant difference, which does not impact the initial maternal folic acid stores and requirements. Estrogen levels have been found to affect lipid metabolism, with male subjects showing more pronounced and consistent results in studies related to disorders of lipid metabolism [36,37]. Therefore, only male offspring were researched in this study. Through observations of serum and hepatic TG levels, and histopathological changes in liver, we found that maternal 5 mg/kg folic acid supplementation during pregnancy effectively ameliorated hepatic steatosis induced by a maternal HFD in 3-week-old offspring.

While DNA methylation is a crucial mechanism frequently invoked to elucidate the influence of folic acid supplementation during pregnancy on the health of offspring, it alone does not provide a comprehensive explanation for the programming effects [32,38]. In recent years, the gut microbiota has been established to be intricately associated with the development of lipid metabolic disorders [39]. The gut microbiota could ameliorate intestinal barrier dysfunction and the propagation of inflammation to organs closely associated with the intestine (such as the liver), affecting its function [40]. The microbial communities from different maternal sources, such as vaginal and breast milk, can impact the establishment of an offspring’s gut microbiota, thereby impacting its disease susceptibility [41,42,43,44,45]. At 3 weeks old, rodent offspring are at a pivotal stage for the development of their gut barrier and the colonization of the gut microbiota [46]. It has been shown that a maternal high-sucrose diet significantly modified the gut microbiota of 3-week-old rat pups, and disrupted their intestinal barrier function, exacerbating hepatic steatosis in adulthood [47]. Furthermore, changes in the gut microbiota have been observed in 3-week-old offspring of mice fed a Western-style diet during pregnancy, and it was revealed that the formation of the gut microbiota during the weaning period is essential for the development of NAFLD in offspring [48]. Our previous study demonstrated the capacity of folic acid to regulate the abundance of gut microbiota [18]. Therefore, we suppose that folic acid supplementation during pregnancy may influence the colonization of the gut microbiota in offspring and consequently have effects on the liver metabolism of offspring. The gut microbiota may serve as another significant mechanism elucidating the intergenerational effects of folic acid.

We conducted sequencing of the colon microbiota in 3-week-old male pups. The diversity analysis revealed that the structure of the gut microbiota in the HFS and CON groups exhibited a higher degree of similarity compared to the differences observed between the HF and CON groups. The administration of folic acid during pregnancy successfully rectified the abundance changes observed in *Firmicutes* and *Bacteroidetes* (including *Bacteroides*) and the *Firmicutes*/*Bacteroidetes* (F/B) ratio in offspring caused by maternal HFD. A previous study has confirmed that *Firmicutes* exhibits superior efficacy over *Bacteroidetes* in enhancing energy absorption, consequently promoting obesity [49]. Consequently, the higher F/B ratios in rodents fed with HFD have been widely accepted. Several studies have suggested that interventions involving plant polyphenols can modulate the F/B ratio and bring about weight loss [50]. Moreover, we observed a reduction in *Desulfobacterota* and *Desulfovibrio* in the offspring of the HFS group. *Desulfobacterota*, an opportunistic pathogenic bacterium, is associated with the pathogenesis of obesity, inflammation, and metabolic disorders [51]. *Desulfobacterota* is capable of producing hydrogen sulfide, which can damage the intestinal barrier integrity and promote the activation of inflammation [52]. *Desulfovibrio*, within the *Desulfobacterota* phylum, has been recognized as a major factor in the production of LPS [53]. Furthermore, correlation analysis showed that microbiota such as *Desulfobacterota* and *Desulfovibrio* were positively correlated with hepatic inflammation-related indices, while microbiota such as *Bacteroides*, *Bacteroidota*, and *Lactobacillus* were negatively correlated with hepatic inflammation-related indices, which are consistent with previous findings [54,55,56]. The results of the gut microbiota suggest that folic acid supplementation during pregnancy ameliorates gut microbiota dysbiosis in male offspring induced by maternal HFD, potentially serving as one of the important factors contributing to the beneficial effects of folic acid on preventing hepatic steatosis in male offspring.

The gut microbiota plays a pivotal role in maintaining the homeostasis of the gut–liver axis [57]. With the disruption of the gut microbiota, bacterial metabolites (such as endotoxins) increase, affecting the integrity of the intestinal barrier [58]. Tight junction proteins, including ZO-1, Claudin 1, and Occludin, are pivotal in ensuring the functionality of the intestinal barrier, thereby serving as indicators of its integrity [59]. Bacterial metabolites are transferred to the liver with the blood when the intestinal barrier is compromised. Within the liver, LPS binding to TLR4 receptor in the liver activates Notch1, causing IκBα to be phosphorylated, resulting in the dissociation of the NF-κB/IκBα complex. The translocation of NF-κB from the cytoplasm to the nucleus facilitates inflammatory factor expression [60,61]. Hepatic inflammation not only contributes to the onset of hepatic steatosis but also exacerbates its progression [62]. In this study, compared to the HF group, the offspring of the HFS group exhibited significant enhancement in colonic mucosal integrity and the upregulation of the expression of tight junction proteins. Maternal folic acid supplementation during pregnancy effectively mitigated the detrimental effects of HFD on the intestinal barrier in the offspring. The improvement in the integrity of the intestinal barrier may lead to a reduction in the translocation of bacterial metabolites into the liver. Therefore, we examined serum levels of LPS in offspring and found that LPS levels were significantly lower in the HFS group. Moreover, we found that folic acid supplementation downregulated the hepatic protein expression levels of TLR4, Notch1, and p-IκBα, consequently inhibiting the nuclear translocation of NF-κB. In short, maternal folic acid supplementation during pregnancy can enhance intestinal barrier integrity in offspring, resulting in LPS leakage reduction and the suppression of liver inflammatory pathway activation.

The inflammatory state of the liver has a substantial impact on lipid metabolism. Numerous studies have confirmed that the overexpression of inflammatory cytokines can inhibit PPAR-α expression and activate SREBP-1c expression [63,64,65]. PPAR-α has been demonstrated to be involved in fatty acid metabolism in various tissues, including the liver, and the activation of PPAR-α leads to significant improvements in hepatic steatosis and inflammation in NAFLD [66]. SREBP-1c can increase the expression levels of lipogenic genes such as FAS and ACC, which enhances the synthesis of fatty acids and accelerates the accumulation of TG [67]. The upregulation of SREBP-1c, along with its downstream genes including FAS and ACC, as well as the downregulation of PPAR-α, are recognized as the interfering factors contributing to the development of hepatic steatosis [68]. In this study, the inhibition of the TLR4/NF-κB inflammatory pathway and reduction in the levels of inflammatory cytokines were followed by the upregulation of PPAR-α and downregulation of SREBP-1c, FAS, and ACC in the livers of 3-week-old male offspring from the HFS group. These findings further explain the observed decrease in serum and liver TG levels, as well as the reduction in hepatic steatosis in the HFS group.

There were some limitations in this study. Firstly, due to the sample limitations, we did not perform Oil Red O (ORO) staining to observe lipid droplets in liver tissues. Although lipid droplet count cannot provide a vivid visualization of lipid droplets, it does enable a quantitative assessment of the extent of hepatic steatosis. Secondly, lard was used as a source of fat, which may produce different results than vegetable fat, because lard exhibits a distinct fatty acid composition and metabolic impact compared to vegetable fats. Thirdly, we gave maternal rats feed containing different doses of folic acid for free consumption, making it difficult to accurately control the intake of folic acid. Fourthly, single nucleotide polymorphisms (SNPs) in the methylenetetrahydrofolate reductase (MTHFR) gene can affect folate metabolism and responsiveness, which may make the results less applicable to individuals with specific genetic characteristics.

## 5. Conclusions

This is the first time it has been demonstrated that folic acid supplementation during pregnancy could alleviate male offspring hepatic steatosis induced by maternal HFD via gut–liver homeostasis. The changes in the gut microbiota composition observed in the 3-week-old male offspring may be attributed to maternal folic acid supplementation during pregnancy, leading to enhanced intestinal barrier integrity. The improved function of the intestinal barrier prevents the entry of endotoxins into the liver, thus inhibiting the expression of the TLR4/NF-κB pathway, mitigating inflammation and the resulting hepatic steatosis. Whether these programming effects endure into adulthood deserves attention in future study.

## Figures and Tables

**Table 1:** Effects of folic acid on the serum parameters.

Groups ^a^	CON	HF	CS	HFS
TG (mmol/L)	0.65 ± 0.06	0.73 ± 0.08 *	0.61 ± 0.07 ^#^	0.59 ± 0.07 ^#^
TC (mmol/L)	0.80 ± 0.091	0.90 ± 0.08	0.81 ± 0.09	0.88 ± 0.10
HDL-C (mmol/L)	0.27 ± 0.03	0.25 ± 0.04	0.27 ± 0.03	0.28 ± 0.04
LDL-C (mmol/L)	0.52 ± 0.04	0.59 ± 0.05 *	0.50 ± 0.06 ^#^	0.53 ± 0.06
GLU (mmol/l)	6.01 ± 0.59	7.07 ± 0.77 *	5.94 ± 0.44 ^#^	6.17 ± 0.64 ^#^
INS (mIU/L)	3.87 ± 0.41	3.87 ± 0.33	3.83 ± 0.36	4.00 ± 0.42
HOMA-IR	1.03 ± 0.11	1.22 ± 0.18 *	1.01 ± 0.10 ^#^	1.09 ± 0.12
LPS (EU/mL)	0.86 ± 0.11	1.21 ± 0.17 *	0.78 ± 0.09 ^#^	0.93 ± 0.12 ^#,†^

^a^ *n* = 10 per group; * *p* < 0.05 vs. CON, ^#^ *p* < 0.05 vs. HF, ^†^ *p* < 0.05 vs. CS. Data are presented as mean ± SD. TG: triglyceride; TC: total cholesterol; HDL-C: high-density lipoprotein cholesterol; LDL-C: low-density lipoprotein cholesterol; GLU: glucose; INS: insulin; HOMA-IR: homeostatic model assessment insulin resistance; LPS: lipopolysaccharide; CON: control group; HF: high fat group; CS: control with folic acid supplementation group; HFS: high fat with folic acid supplementation group; SD: standard deviation.

## Data Availability

The datasets used and/or analyzed during the current study are available from the corresponding author upon reasonable request.

## Supplementary Materials

The following supporting information can be downloaded at: https://www.mdpi.com/article/10.3390/nu15224726/s1, Table S1: Composition of the diets (g/kg diet).
