# Supplementary material for: Maternal Folic Acid Supplementation during Pregnancy Prevents Hepatic Steatosis in Male Offspring of Rat Dams Fed High-Fat Diet, Which Is Associated with the Regulation of Gut Microbiota
Source: Nutrients. 2023 Nov 8;15(22):4726. doi: 10.3390/nu15224726 (PMC10675082; doi:10.3390/nu15224726)
Supplement: Supplementary file 1 [file nutrients-15-04726-s001.zip › nutrients-2658352-supplementary.pdf]

Table S1 Composition of the diets (g/kg diet).

Dietary ingredient	Type of diet			
	CON	HF	CS	HFS
Casein	189.57	258.46	189.57	258.46
Corn starch	479.81	-	479.81	-
Maltodextrin	118.48	161.54	118.48	161.54
Sucrose	65.21	88.91	65.21	88.91
Cellulose	47.39	64.52	47.39	64.52
Lard	18.96	316.62	18.96	316.62
Soybean oil	23.70	32.21	23.70	32.21
Cystine	2.84	3.88	2.84	3.88
DIO	9.48	12.92	9.48	12.92
Multivitamins (except folic acid)	9.48	12.92	9.48	12.92
Choline	1.90	2.58	1.90	2.58
Calcium carbonate	5.21	7.11	5.21	7.11
Dicalcium phosphate	12.32	16.80	12.32	16.80
Potassium citrate monohydrate	15.64	21.32	15.64	21.32
Folic acid	0.002	0.002	0.005	0.005
Total energy (kcal/kg diet)	3850	5243	3850	5243

CON, control; HF, high-fat; CS, control with folic acid supplement; HFS, high-fat with folic acid supplement.
